# The ENCCA-WP7/EuroSarc/EEC/PROVABES/EURAMOS 3rd European Bone Sarcoma Networking Meeting/Joint Workshop of EU Bone Sarcoma Translational Research Networks; Vienna, Austria, September 24–25, 2015. Workshop Report

**DOI:** 10.1186/s13569-016-0043-5

**Published:** 2016-03-16

**Authors:** Leo Kager, Jeremy Whelan, Uta Dirksen, Bass Hassan, Jakob Anninga, Lindsey Bennister, Judith V. M. G. Bovée, Bernadette Brennan, Javier M. Broto, Laurence Brugières, Anne-Marie Cleton-Jansen, Christopher Copland, Aurélie Dutour, Franca Fagioli, Stefano Ferrari, Marta Fiocco, Emmy Fleuren, Nathalie Gaspar, Hans Gelderblom, Craig Gerrand, Joachim Gerß, Ornella Gonzato, Winette van der Graaf, Stefanie Hecker-Nolting, David Herrero-Martín, Stephanie Klco-Brosius, Heinrich Kovar, Ruth Ladenstein, Carlo Lancia, Marie-Cecile LeDeley, Martin G. McCabe, Markus Metzler, Ola Myklebost, Michaela Nathrath, Piero Picci, Jenny Potratz, Françoise Redini, Günther H.S. Richter, Denise Reinke, Piotr Rutkowski, Katia Scotlandi, Sandra Strauss, David Thomas, Oscar M. Tirado, Franck Tirode, Gilles Vassal, Stefan S. Bielack

**Affiliations:** Department of Paediatrics, St. Anna Children’s Hospital, Medical University Vienna, Vienna, Austria; Children’s Cancer Research Institute, Vienna, Austria; University College London Hospitals, London, UK; Universitätsklinikum Münster, Munster, Germany; Oxford University, Oxford, UK; Leids Universitair Medisch Centrum, Leiden, The Netherlands; Sarcoma Patients Euronet, Sarcoma UK, London, UK; Central Manchester University Hospitals, Manchester, UK; Hospital Universitario Virgen del Rocío, Seville, Spain; Gustave-Roussy Cancer Campus, Villejuif, France; York, UK; Centre Léon Bérard, Lyon, France; Regina Margherita Children’s Hospital, Turin, Italy; Istituto Ortopedico Rizzoli, Bologna, Italy; Radboud University Medical Centre, Nijmegen, The Netherlands; Newcastle upon Tyne Hospitals, Newcastle, UK; Sarcoma Patients Euronet, Udine, Italy; Institute of Cancer Research, London, UK; Klinikum Stuttgart-Olgahospital, Stuttgart, Germany; Sarcoma Research Group, Molecular Oncology Laboratory, Institut d’Investigació Biomèdica de Bellvitge (IDIBELL), L’Hospitalet de Llobregat, Barcelona, Spain; University of Manchester, Manchester, UK; Universitätsklinikum Erlangen, Erlangen, Germany; Oslo University Hospital, Oslo, Norway; Technische Universität München, Munich, Germany; Klinikum Kassel, Kassel, Germany; Université de Nantes, Nantes, France; Sarcoma Alliance through Research and Collaboration, Ann Arbor, USA; Maria Sklodowska-Curie Memorial Cancer Centre, Warsaw, Poland; Garvan Institute of Medical Research, Darlinghurst, Australia; Institut Curie, Paris, France

**Keywords:** Osteosarcoma, Ewing sarcoma, Bone sarcoma, Immunotherapy, Next generation sequencing, Pharmacogenomics, Translational research

## Abstract

This report summarizes the results of the 3rd Joint ENCCA-WP7, EuroSarc, EEC, PROVABES, and EURAMOS European Bone Sarcoma Network Meeting, which was held at the Children’s Cancer Research Institute in Vienna, Austria on September 24–25, 2015. The joint bone sarcoma network meetings bring together European bone sarcoma researchers to present and discuss current knowledge on bone sarcoma biology, genetics, immunology, as well as results from preclinical investigations and clinical trials, to generate novel hypotheses for collaborative biological and clinical investigations. The ultimate goal is to further improve therapy and outcome in patients with bone sarcomas.

## Organization

The 3rd European bone sarcoma networking meeting was held at the Children’s Cancer Research Institute in Vienna, Austria on September 24–25, 2015. It was organized by Stefan Bielack, Stuttgart, and Leo Kager, Vienna, and supported by the European Network for Cancer research in Children and Adolescents Work Package 7 (ENCCA-WP7, represented by Stefan Bielack), the EUROpean Clinical Trials in Rare SARComas initiative (EuroSARC, represented by Bass Hassan, Oxford), the European Ewing Consortium (EEC, represented by Jeremy Whelan, London), the PROspective VAlidation of Biomarkers in Ewing Sarcoma network (PROVABES, represented by Uta Dirksen, Münster), and the European branch of the EURopean and AMerican Osteosarcoma Study Group (EURAMOS, represented by Stefan Bielack and Jeremy Whelan).

## High-grade osteosarcoma

### Genomics

High-grade osteosarcomas (HGOS) have complex karyotypes showing abundant structural and numerical aberrations. Michaela Nathrath, Kassel, presented data from the Cooperative Osteosarcoma Biology Study Group. Exomes of 31 HGOS were sequenced and their evolutionary landscape was deciphered by inferring clonality of the individual mutation events. Exome findings were interpreted in the context of mutation and single-nucleotide polymorphism (SNP) array data from a replication set of 92 tumours. 14 genes were identified as the main drivers, some of which were formerly unknown in the context of HGOS. Tumour protein 53 (TP53) and molecular pathways functionally similar to TP53 seem to drive genomic instability in HGOS. More than 80 % of HGOSs exhibited a specific combination of single base substitutions, loss of heterozygosity (LOH), or large-scale genome instability signatures characteristic of breast cancer 1/2, and early onset (BRCA1/2)-deficient tumours. The findings imply that multiple oncogenic pathways drive chromosomal instability during osteosarcoma evolution and result in the acquisition of BRCA-like traits, which could be therapeutically exploited.

The Norwegian Sarcoma Consortium (http://NoSarC.org), a national collaboration collecting samples from all sarcoma patients in Norway for next generation sequencing (NGS)-based analysis and the search for new therapies, was introduced by Ola Myklebost, Oslo. He presented preclinical studies of a panel of osteosarcoma cell lines using NGS of DNA and RNA (Ref Lorenz et al. PMID 26672768). They found hundreds of fusion transcripts, of which only a small fraction corresponded to fusion genes, suggesting a phenotype of trans-splicing, by which transcripts from different genes are joined during splicing. They did, however, find a recurrent fusion gene involving *PMP22* (which encodes peripheral myelin protein 22) and *ELOVL5* (which encodes ELOVL fatty acid elongase 5). Whereas only a few of the genomic fusions produced fusion transcripts, the trans-spliced mRNAs should yield large numbers of neoantigens that could support immune checkpoint therapies. Furthermore, all cell lines had completely abrogated the p53 response, largely caused by copy number-neutral fusions and aberrations of *TP53*. The group will perform the NGS analysis of the SARC028 trial of Pembrolizumab in sarcoma.

### Modulation of drug effects in osteosarcoma cells, drug resistance and pharmacogenomics

Given the plateau in survival over the last three decades, Anne-Marie Cleton-Jansen, Leiden, discussed new opportunities for HGOS therapy. Improving the efficacy of chemotherapy might provide one such opportunity and could be achieved, for example, by modulating the pharmacodynamic effects of drugs in HGOS cells. Using a small interfering RNA (siRNA) screen, the aven-ATM serine/threonine kinase-checkpoint kinase 1 (ATM-CHEK1) pathway was identified as a target to sensitize osteosarcoma cells to conventional chemotherapeutic agents. Modulation of Aven-ATM-CHEK1 may also provide a novel strategy towards improving HGOS therapy.

Aurélie Dutour, Lyon, demonstrated that chemotherapeutic treatments trigger an increase of netrin-1 (NTN1) expression, accompanied by an increase in netrin-1 dependence receptors DCC and UNC5H, and that combining chemotherapeutic agents and netrin-1 interference potentiates cancer cell death. The effect of anti-Ntn1 antibody combined with doxorubicin was investigated in an orthotopic metastatic rat model of osteosarcoma. The combination slows down osteosarcoma progression, significantly prolongs animal survival and prevents metastatic dissemination. Therefore, combining conventional drugs with Ntn-1 interference could lead to superior efficacy as well as lower chemotherapy doses for HGOS treatment.

Blocking mammalian target of rapamycin (mTOR) activity is a promising approach to the treatment of patients with HGOS, although mTOR monotherapy has met with mixed results. Winette van der Graaf, London, suggested that combination therapy might be the key to success. Using two in vivo osteosarcoma models, she and her colleagues demonstrated that the activity of the mTOR-inhibitor temsirolimus is significantly enhanced by the addition of either cisplatin or bevacizumab. Moreover, extensive immunohistochemical and 3′-deoxy-3′-^18^F-fluorothymidine positron emission tomography/computed tomography (^18^F-FLT-PET/CT) analyses of tumour response indicated that tumour volumes underestimated treatment efficacy, and that ^18^F-FLT-PET/CT can potentially be used to measure response in the early phases of treatment. These findings suggest the need for further exploration of temsirolimus combined with either cisplatin or bevacizumab for HGOS, with the incorporation of ^18^F-FLT-PET scans.

Stefano Ferrari, Bologna, pointed out that novel approaches to the treatment of HGOS should include patient risk stratification. For example, over-expression of the ATP-binding cassette transporter B1 (ABCB1) at diagnosis is involved in the processes of resistance to classic antineoplastic drugs, identifying patients with a poor prognosis in Italian sarcoma group (ISG) studies. The ISG and Spanish sarcoma group are presently carrying out a trial (ISG/OS-2, ClinicalTrials.gov number: NCT01459484) in which patients are stratified into different regimens based on ABCB1 expression. Besides ABCB1, overexpression of the DNA excision repair protein ‘excision repair cross-complementation group 1’ (ERCC1) was found to be associated with a high relapse rate and poor EFS and OS. The co-evaluation of ERCC1 and ABCB1 protein expression showed that patients positive for both markers had a significantly worse prognosis.

Pharmacogenomics, which aims to tailor drug therapy based on the genomic ‘make-up’ of normal host cells and cancer cells, can help to improve drug therapy. Accordingly, there has been an increase in pharmacogenomic investigations in patients with HGOS. Dr. Ferrari pointed out that a special effort should be made to prospectively undertake pharmacogenetic profiling of patients entering clinical trials.

### From bench to bedside, and back to bench

The beneficial effect of combining chemotherapy with the bisphosphonate zoledronate in syngenic models of rat osteosarcoma constituted the rationale for the French OS2006 trial (NCT00470223). Laurence Brugières, Villejuif, reported on the final results of the OS2006 trial conducted in France between 2007 and 2014. 315 patients with HGOS (83 % localised and resectable) were randomised to receive or not 10 injections of zoledronate in addition to chemotherapy. The final analysis of this trial showed that the addition of zoledronate to pre- and post-operative chemotherapy did not improve event-free and overall survival of patients with previously untreated osteosarcoma. This analysis also showed a slight excess of events and deaths in patients treated with zoledronate as compared to patients without zoledronate, leading to a non-significant difference in EFS and OS.

Françoise Rédini, Nantes, provided information on how the French group will explore the biological background for the unexpected results of the OS2006 trial. Several hypotheses have been raised which include involvement of infiltrating macrophages, receptor activator of nuclear factor-kappa B (RANK)-expressing osteosarcoma cells, tartrate-resistant acid phosphatase (TRAP) expressing cells in the tumour bone niche, and the impact of the hormone microenvironment. The objective of ancillary studies performed on OS2006 biological samples is to determine whether the results are linked to the disease itself (HGOS), to the bone microenvironment, or to the age of the patients.

### Immunology and immunotherapy in HGOS

David Thomas, Darlinghurst, provided an overview of immunotherapeutic approaches, which hold great promise for cancer treatment. HGOS is noted to be an immunologically interesting disease, for multiple reasons. For example, HGOS is assumed to derive from osteoblasts, a unique cell type that, together with the osteoclast, plays an important role in bone development and physiology. Dr. Thomas reviewed the complex signalling molecules and interdependencies that link the osteoblast and osteoclast, and their overlap with the immune system. There is an association between mutational load and tumour neo-antigens, apparently important to the effectiveness of a new generation of immunotherapies (e.g., immune checkpoint inhibitors). In addition, HGOSs are noted to be genotypically complex and unstable, potentially making them good targets for immunotherapies. There is a long history of immunological approaches to HGOS (e.g., muramyl-tripeptide and interferons), which reinforces optimism about their susceptibility to new developments in immune therapies.

Piotr Rutkowski, Warsaw, showed that overexpression of programmed cell-death ligand 1 (PD-L1) on tumour cells, which interact with PD-1 on cytotoxic T-lymphocytes, impedes antitumour immunity and results in immune evasion. Interruption of the PD-L1/PD-1 pathway therefore represents an attractive therapeutic strategy to reinvigorate tumour-specific T cell immunity. He reported that PD-L1 had been observed to be significantly expressed in different subtypes of sarcomas including osteosarcoma (36 %), leiomyosarcoma (97 %) and Ewing sarcoma (39 %), while Kim et al. detected PD-L1 expression in 70 % of leiomyosarcomas, 67 % of Ewing sarcomas and 75 % of synovial sarcomas. These levels of expression, together with the poorer outcome of sarcoma patients with PD-L1 positivity, justify further exploration of the role of PD-L1 antibody in the treatment of sarcomas. Currently, the Soft Tissue and Bone Sarcoma Group of the European Organisation for Research and Treatment of Cancer (EORTC) and several pharmaceutical companies are planning phase II studies of anti-PD-L1 antibodies in advanced sarcomas, including HGOS.

The focus of the immunoSARC project, presented by Javier Martin-Broto, Sevilla, is to explore immunomodulation as a therapeutic approach in sarcomas. The perspective is not only to investigate the impact of immune target drugs (e.g., PD-1 or PD-L1 inhibitors), but also to focus on the role of immunomodulation via combinations of these drugs with anti-angiogenic agents. Whereas the link between angiogenesis of tumours and immune evasion (e.g., via vascular endothelial growth factor A [VEGFA]) is well known, this connection has been poorly studied in sarcomas even though some tumours are highly vascularised. A “pick the winner” randomized phase II trial has been designed, comparing the anti-PD-1 antibody nivolumab alone versus the combination of nivolumab plus pazopanib (a multi-targeted tyrosine kinase inhibitor that blocks angiogenesis) in cohorts of patients with bone sarcomas and soft tissue sarcomas. This investigation aims to explore whether combination arms prove to be synergistic in efficacy in selected sarcoma subtypes and if some immune targets (e.g., PD-1/PDL-1) could be predictive at least in some sarcoma subtypes.

Franca Fagioli, Torino, provided preclinical data on two different adoptive immunotherapy strategies for the treatment of sarcomas, including HGOS, namely human leukocyte antigen (HLA)-unrestricted and HLA-restricted strategies, with cytokine-induced killer (CIK) cells and sarcoma-specific cytotoxic T-lymphocytes (CTLs), respectively. The results of her investigations have provided preclinical proof-of-concept for an effective strategy to attack sarcomas with CIKs. A Phase I study with CIKs for patients with high-risk sarcomas, including HGOS, is being prepared.

Bass Hassan, Oxford, reviewed current knowledge on the macrophage activating drug mifamurtide in the context of osteosarcoma and provided an overview on the MEMOS (‘A mechanistic study of mifamurtide in patients with metastatic and/or recurrent osteosarcoma’) trial (EudraCT Number: 2012-000615-84; http://www.oncology.ox.ac.uk/trial/memos) of the EuroSARC group (http://eurosarc.eu/). The primary objective of this EuroSARC funded investigator initiated trial is to analyze biological effects of mifamurtide, and so requires tumour biopsies. A number of centres in UK, Norway, Italy and the Netherlands have begun recruiting patients.

## Chondrosarcoma and spindle/pleomorphic bone sarcomas

Judith Bovée, Leiden, provided an update on the biology of chondrosarcoma (CS). A novel mouse model for peripheral CS has emphasized that, in addition to biallelic inactivation of exostosin glycosyltransferase (*EXT1* or -*2*) in chondrocytes, additional alterations affecting either the TP53 or cyclin-dependent kinase inhibitor 2A (CDKN2A) cause malignant transformation towards secondary peripheral chondrosarcoma. It was shown that mutations in isocitrate dehydrogenase (*IDH1* or -*2*) are an early event for central tumours: the oncometabolite D-2-hydroxyglutarate induced by the mutation inhibits osteogenic differentiation and promotes chondrogenic differentiation, causing enchondromas of bone. In central chondrosarcomas, the *IDH* mutation is, however, no longer a driver mutation, as inhibition of the mutant protein has no effect on the tumourigenic properties of chondrosarcoma cell lines. Other additional genetic alterations are instead involved, affecting amongst others the TP53 and retinoblastoma (Rb) pathways, as well as collagen, type II, alpha 1 (COL2A1), neuroblastoma RAS viral oncogene homolog (NRAS), and other signalling pathways (e.g., hedgehog, mTOR, Bcl-2, survivin).

Jeremy Whelan, London, discussed treatment of mesenchymal CS, which is a very rare subtype accounting for approximately 5 % of chondrosarcoma. The diagnosis is supported by identification of a translocation involving the transcription factor *HEY1* and the nuclear receptor coactivator 2 (*NCOA2*). It mostly affects young adults and can arise at multiple anatomical sites, either in soft tissue or bone. A recent publication by Frezza et al. of a case series derived from the experience of European centres of excellence, has supported the prognostic significance of metastatic disease and proposed a survival advantage for the use of adjuvant chemotherapy. A second recent publication by Xu et al. using data extracted from subsets of patients reported in multiple retrospective series, drew opposite conclusions. Examples of where observational data had influenced clinical practice, but were refuted by data from randomised trials, were used to emphasize the importance of randomised studies, even in very rare sarcoma subtypes. The challenges of undertaking a randomised study of chemotherapy in mesenchymal chondrosarcoma were described using a recently published typology for access to trials and were concluded to be too formidable.

Hans Gelderblom, Leiden, reported on the COSYMO Study (EudraCT No: 2013-005155-32) in which three cohorts of patients (i.e., conventional chondrosarcoma, dedifferentiated/mesenchymal CS, and myxoid/round cell liposarcoma [MLS]) are treated with a combination of mTOR inhibition and cyclophosphamide. The rationale to use this drug combination in patients with CS and MLS derives from preclinical and clinical investigations.

Piero Picci, Bologna, stressed the importance of a more accurate classification for spindle/pleomorphic bone sarcomas, as recently drawn up for soft tissue sarcomas. This would permit more specific chemotherapy regimens for the different entities within collaborative studies, made necessary by the rarity of these sarcomas.

## Ewing sarcoma

### Biology of Ewing sarcoma and Ewing-like sarcomas

Heinrich Kovar, Vienna, reported on novel players in the pathogenesis of Ewing sarcoma (EwS). *EWS*-*FLI1* affects a number of nicotinamide adenine dinucleotide (NAD) metabolizing enzymes, resulting in reduction of NAD levels in EwS cells. Drugs, which interact with the NAD metabolome (e.g., nicotinamide phosphoribosyltransferase [NAMPT]) may be considered for preclinical development in EwS.

David Herrero-Martin, Barcelona, provided data on non-coding RNAs in EwS. Analysis of the EwS methylome showed miR-10a (5p) hypermethylation; and miR-10a was found to be expressed at very low levels in both EwS cell lines and patient samples. Reintroduction of miR-10a in two EwS cell lines reduced migratory capacity and decreased clonogenic growth. An iTRAq (isobaric tags for relative and absolute quantification) proteomic analysis of miR-10a transfected cells allowed identification of several proteins that could be related to the described phenotype.

Oscar Martinez Tirado and co-workers, Barcelona, revealed a role for ephrin A receptor 2 (EphA2) in the progression of EwS. This receptor chiefly participates in the migratory capacity of EwS cells by ligand-independent means. Work is in progress to decipher the molecular mechanisms associated with such effects.

Franck Tirode, Paris, reported that four Ewing-like sarcoma entities have been described as small round cell tumours presenting morphological characteristics of EwS but carrying a different chromosomal translocation (either one of *BCOR*-*CCNB3*, *CIC*-*DUX4*, *EWSR1*-*NFATc2* or *EWSR1*-*PATZ1*). The resemblance of these Ewing-like tumours, both to ES and among each other, was examined. It was first demonstrated that EwS presenting rare fusion variants such as *FUS*-*ERG* or *FUS*-*FEV* are transcriptionally undistinguishable from classical *EWSR1*-*FLI1* or *EWS*-*ERG* positive tumours. Second, expression profiling and careful examination of clinical and pathological data indicated that the four Ewing-like sarcomas are far from resembling EwS and are distinct entities. Tirode et al. proposed to consider EwS as tumours carrying only fusions involving FET family proteins (*EWSR1* or *FUS*) with ETS transcription factor genes (*ERG* and *PEA3* types). While *CIC*-*DUX4* and *BCOR*-*CCNB3* are now classified as undifferentiated/unclassified sarcomas in the latest WHO sarcoma classification, said group proposed adding to this category all other types of Ewing-like translocations, as *EWSR1*-*NFATc2* and *EWSR1*-*PATZ1*. Finally, it might even be advisable to provide a different nomenclature for Ewing-like sarcomas such as small round cell sarcoma with *X*-*X* translocation.

### Epigenetics, inhibition of signalling pathways and circulating tumour DNA in EwS

Günther Richter, Munich, reported that next-generation sequencing (NGS) data confirm *EWS/ETS* translocations as the crucial driver event of EwS tumourigenesis. *EWS/FLI1* induces altered epigenetic marks, and targeting ‘epigenetic readers,’ such as bromodomain-containing proteins (e.g., BRD3/4) in EwS, reduces *EWS/FLI1* expression. Treatment with bromodomain and extra-terminal motif (BET) inhibitors such as JQ1 blocks the typical expression profile of EwS. Moreover, JQ1 inhibits proliferation, induces apoptosis and reduces EwS tumour growth. When combined with phosphatidylinositol-4,5-bisphosphate 3-kinase (PI3K) inhibitor BEZ235, inhibition of BRD3/4 may offer new opportunities for combination therapy in EwS.

Emmy Fleuren, Nijmegen, focused on the topic of multi-receptor tyrosine kinase targeting in EwS. Unfortunately, inhibitors targeting a single receptor tyrosine kinase (RTK), such as insulin-like growth factor 1 receptor (IGF-1R), have been shown to be insufficient for EwS treatment in clinical practice. Since research indicates that tumours often do not rely on a single RTK and different RTKs can compensate for one another to maintain tumour growth, Fleuren et al. hypothesized that targeting multiple RTKs at once may be a more promising approach. They therefore investigated the co-expression patterns and co-targeting effects of various oncogenic RTKs implicated in EwS in 30 primary ES patient samples. Significant, strong positive correlations and co-expression patterns were observed between IGF-1R and MET, IGF-1R and AXL, and MET and AXL receptors. Co-targeting these RTKs was synergistic or at least additive in 6/6 EwS cell lines in vitro, with the most pronounced effects in IGF-1R-targeted combinations, indicating that IGF-1R deserves further investigation, particularly in combinations.

Jenny Potratz, Münster, provided data showing that the receptor tyrosine kinase (RTK) ‘recepteur d’origine nantais*’* (RON) is expressed and active in EwS, with over-expression in tumours derived from metastatic disease. Evidence of function in pro-metastatic cellular features supports RON as a potential therapeutic target. However, targeting strategies are challenged by the isoform known as short-form RON which lacks the extracellular antibody-binding domain, thereby bypassing antibody inhibition. This exemplifies that isoforms, principally described in diverse RTKs, require attention and further understanding to adapt targeting strategies.

Bass Hassan discussed the rationale for dual inhibition of IGF-1R and insulin receptor (IR) in EwS. Moreover, he presented the LINES trial (http://www.oncology.ox.ac.uk/trial/lines): a Bayesian single-arm phase II trial in which the dual anti-IGF-1R/IR drug linsitinib is investigated in patients with relapsed and/or refractory EwS. This EuroSARC biomarker directed study is currently recruiting in UK, Italy, Germany and the Netherlands, despite prolonged administrative and set up delays. The study incorporates intensive analysis including ^18^FDG-PET-CT and biopsies (pathway and gene expression responses including RNASeq) before and after high dose linsitinib exposure, and aims to discover potential mechanisms of dual IGF1R/IR kinase sensitivity and resistance.

Katia Scotlandi, Bologna, reported on the interaction between the cytotoxic drug trabectedin (Yondelis^®^) and IGF-1 signalling. In EwS, trabectedin may not only inhibit but also enhance the binding of *EWS*-*FLI1* to certain target genes, leading to upregulation of IGF1R. Combination of trabectedin and anti-IGF-1R inhibitors represents a potential therapeutic option for patients with EwS.

Markus Metzler, Erlangen, provided information on the EFACT (EWS-FLI sequence analysis from ctDNA) project, which assesses circulating tumour DNA (ctDNA) as a response marker in EwS. EFACT is implemented as an accompanying research project to the ongoing EWING2008 trial. Extended application to relapsed EwS appears particularly informative considering that personalized treatment schedules are often necessary in this cohort. Analysis of ctDNA copy numbers is suited for integration in preclinical studies and Phase I/II trials as an additional response marker.

## Delivering trials effectively for bone sarcoma

### What can be learned from recent sarcoma trials?

Hans Gelderblom provided an overview on recently published clinical trials in HGOS, EwS and CS and discussed the difficulties in conducting investigator-initiated clinical trials in bone sarcomas. Collaboration, creation of centres of expertise for bone sarcomas, innovative statistical designs, lowering administrative burden, inventive funding, molecular tumour boards, etc. might help to boost investigator-initiated research in bone sarcomas.

Marie-Cecile LeDeley, Villejuif, summarized results from the EURO-EWING 99 trial (NCT00020566) and discussed how to make international cooperation more efficient, based on the experience acquired in the EURO-EWING 99 trial.

Uta Dirksen, Münster, provided an update on the international EWING 2008 trial (NCT00987636; https://www.clinicaltrialsregister.eu/ctr-search/trial/2008-003658-13/DE) which is open in 11 countries and more than 100 centres. The trial includes three arms: standard risk, high risk and very high risk with different randomised questions. It is the first clinical trial to also include a randomised question for patients with very high risk EwS with primary disseminated disease. An interdisciplinary international tumour board is open for patients treated within the clinical trial and also for patients who do not have the chance to receive treatment within clinical trials. Part of the report focused on the administrative barriers to opening an investigator-initiated trial in Europe with national directives on top of the EU directives, and the advantages of a harmonized online registration and data entry system (MARVIN; https://www.xclinical.com/en/products/marvin), now used by many European clinical trials.

The “Prospective Validation of Biomarkers in EwS for a personalized medicine” project (PROVABES, http://provabes.uni-muenster.de) was also presented. Provabes is a joint proposal by the leading European Ewing sarcoma study groups and basic and translational researchers. Aim of the study is to validate previously described putative predictive and prognostic biomarkers. The group expects that a combination of markers rather than a single biomarker will provide the most informative prognostic algorithm for patients with EwS.

A short summary was provided on the long-term follow up and late effect study, PanCareLife (http://pancarelife.eu), focusing on fertility after cancer.

Bernadette Brennan, Manchester, reported on the Euro Ewing 2012 trial (EudraCT Number 2012-002107-17, http://www.euroewing.eu/clinical-trials/ee2012-trial) which has been opened and recruiting patients since December 2013 in UK, Spain and France. Thus far, 54 European centres are open with plans to set up centres in Australia and Israel in 2016. The opening of all international centres, sponsored by the Birmingham ‘Cancer Research UK Clinical Trial Unit’ (CRUK CTU), involving each country’s interpretation of EU trials regulations, has not been quick or easy. The CTU should perhaps have considered that protracted contracts with other countries would occur and have avoided the ensuing delays by addressing the problem much earlier. The presentation of data using the same investigational medical product (IMP) in another bone tumour has caused concern among investigators and may affect recruitment. Lastly the ‘R3’ cohort of EwS were not included in this trial and every effort must now be made by EEC to rectify this, considering that their prognosis remains dismal.

Martin McCabe, Manchester, reported on the ‘rEECur’ trial (http://www.euroewing.eu/clinical-trials/reecur/index), which aims to compare four commonly used chemotherapy regimens for efficacy and toxicity in refractory and relapsed EwS. It utilises a multi-arm, multi-stage trial design to exclude less active and/or more toxic arms at an early stage, and will therefore avoid recruitment of large numbers of patients to these less active, more toxic chemotherapy regimens. rEECur represents the most extensive collaboration yet between European Ewing sarcoma clinical trial groups. This collaboration has been facilitated by the acquisition of European Commission funding. However, despite a keen willingness to collaborate and a desire to successfully answer the study questions, opening the study has been hampered by the requirement of each EU member state to individually interpret the EU Clinical Trials Directive through its Competent Authority, and to assess the ethical issues of the study. Two years into a 5-year grant, the study is open in six out of the planned thirteen countries. None of the queries raised by Competent Authorities or ethics committees have yet resulted in any changes to the design, to implementation of the study protocol or to the patient consent process, but the bureaucracy required to repeat the process in thirteen countries has contributed significantly to the time and resources needed to open the study. Major European funders should consider this time delay as an inevitable component of cross-border European clinical trials when allocating resources for running international trials.

Stefano Ferrari commented on two ES trials run by ISG and the Scandinavian Sarcoma Group (SSG). ISG/SSG3 (1999–2006) enrolled 300 patients with non metastatic EwS, and ISG/SSG4 (1999–2008) enrolled 102 with metastatic EwS. Both protocols envisaged the use of high-dose chemotherapy (HDCHT) and peripheral blood stem cell (PBSC) support for high-risk patients (poor responders to induction chemotherapy or metastatic patients). Patients with non-metastatic EwS, responding poorly to induction treatment with the VACA-IE regimen, achieved the same probability of event-free survival as good responders, thanks to the addition of HDCHT. Surgery was the main option for local control. The use of postoperative radiotherapy should be provided in cases of inadequate surgical margins and when the histological response does not affect local control. Females and very young paediatric patients experience higher bone marrow toxicity. In patients with synchronous metastases (lung or only one bone metastasis) a 5-year overall survival rate of 50 % can be achieved with aggressive treatment with HDCHT and total lung irradiation. Complete radiological response of lung metastases is predictive of survival. Post-relapse survival is very poor in recurrent patients in spite of intensive chemotherapy treatment with high-dose ifosfamide and high-dose busulfan and melphalan. Second complete remission is the main factor predicting post relapse survival. The role of HDCHT in this subset of patients is uncertain.

Sandra Strauss, London, reported on the Sarcoma Research through Collaboration SARC025/SP1 trial (http://sarctrials.org/SARC025), which is a collaborative US/European phase I study of a combination of the poly(ADP-ribose) polymerase (PARP) inhibitor, Niraparib, with Temozolomide in EwS. Dr. Strauss commented on the regulatory challenges that have arisen as the sponsor of the trial (the US SARC group) is not based in the European Economic Area (EEA). Models that may help to overcome such obstacles were discussed.

Stefan Bielack commented on similarities between the experiences of clinical osteosarcoma trialists with those reported in relation to Ewing sarcoma trials. He used the EURAMOS-1 study of the European and American Osteosarcoma Study Group as an example to describe how the multiple challenges arising during the past one and a half decades of Pan-European and transatlantic cooperation were addressed. He emphasized the need for constant communication within a consortium of this size, the benefits of joint training activities for site staff, the need to follow common predefined rules as well as to find compromises where necessary. The EURAMOS consortium managed to accomplish the primary objectives of its intergroup trial, and to use its visibility to lobby for a better European environment for investigator-initiated clinical trials.

### Statistical issues

Joachim Gerß, Münster, commented on how statistics can contribute to making bone sarcoma research more effective. In the face of new discoveries or information collected in the course of a trial, the adoption of adaptive clinical trial designs enables flexible adjustments to be made, such as in sample size or target population. Flexible-adaptive clinical trial designs usually adhere to established quality criteria, as the meaning of type I error, in marked contrast to the completely different Bayesian statistical approach. The Bayesian statistical approach allows prior knowledge to be included, enabling the amount of information utilized in statistical analyses to be increased. The knowledge resulting from statistical evaluation is also outlined very clearly.

Carlo Lancia, Jakob Anninga, and Marta Fiocco, Leiden, discussed multi-drug regimens and dose intensity in HGOS, focusing on the decision-making process of dynamically adjusting therapy based on toxicity. Toxicity is a time-dependent confounder since it is both a mortality risk-factor and a predictor of subsequent exposure to cytotoxic agents. In the presence of time-dependent confounders, the classical Cox model yields biased estimates of hazard ratios for the variables being investigated. Marginal structural Cox models are a possible solution to this problem.

### How to construct and perform informative bone sarcoma trials?

Nathalie Gaspar, Villejuif, a member of the Innovative Therapies for Children with Cancer (ITCC) consortium, discussed strategies to prioritize agents for further development in bone sarcoma. In a world where an increasing number of new drugs with increasingly varied mechanisms of action are being developed each year, international networks should think about strategies to prioritize agents for further development in bone sarcoma, along four axes. (1) To define appropriate drugable targets with relevant bone sarcoma biology at diagnosis and relapse. (2) To ensure strong preclinical evidence of drug efficacy alone and in combination in multiple bone sarcoma models, taking into account the microenvironment (bone, neoangiogenesis, immune system). (3) To promote drugs with appropriate toxicity profiles that could be compatible with relapse but mainly with first-line chemotherapy regimens. (4) To anticipate when to introduce the drug according to this mechanism of action, such as on bulky tumour or in minimal residual disease. Collaboration among clinicians and biologists but also statisticians is crucial, as the main objective remains to improve patient outcome by introducing new drugs into first-line treatment of bone sarcomas at whatever age,.

Ornella Gonzato, Udine, a representative of SPAEN (Sarcoma Patients EuroNet; http://www.sarcoma-patients.eu), discussed the efforts made over the last few years, by both the scientific and patient communities in the field of rare cancers, to allow patients access to promising new experimental drugs. Clinical studies on rare tumours, as bone sarcomas, require a significant collaborative network, resources and sufficient numbers of patients to lend statistical significance and value to study outcomes. As these tumours are, by definition, rare, the problem is the scarcity of the numbers involved. It therefore follows that as many patients as possible must be recruited. She outlined the crucial role that correct information always plays in patient recruitment. This means managing information by defining what, how, where and by whom it should be delivered to patients, in order to make them aware of the nature of clinical trials and the benefits of participating in one of them.

Lindsey Bennister, London, a charity representative, stated that patients expect an appropriate trial to be suggested by their clinician as part of their treatment plan. The National Cancer Patient Experience Survey (England, 2014) found that only 35 % of sarcoma patients were asked by their clinician to participate in research. However, 64 % of these went on to participate, showing that patients are interested and willing to take part when invited to do so by their clinician. She provided recommendations on how to improve information on trials to patients. This included raising awareness about the importance of recommending trials to patients through a European campaign targeted at clinicians and supported by charities, patient groups and research networks. It also entailed setting up a one-stop sarcoma portal for clinical trials (in Europe), rather than having multiple places to search. Moreover, it was emphasised that clinical trials should get smarter about ‘marketing’ themselves in order to attract and retain participants, e.g., by sharing the excitement of what difference the trial could make in the future and clearly setting out the benefits to individual patients.

In his capacity as a consumer representative, Christopher Copland, York, presented a network of groups and individuals from across Europe (http://unite2cure.org), calling for better treatment and better access to therapy for children and young people with cancer. The network decided to take the opportunity of Childhood Cancer Awareness month to build support for bringing about reform of Paediatric Medicines Regulations (2007). They set the target of collecting 1000 signatures for an e-petition by the end of September 2015, a goal which they easily exceeded. Amongst its supporters their website now lists many prominent professionals and numerous well-respected organisations in the field. They carried the initiative forward at meetings with the Cancer Drugs Development Forum and the European Medicines Agency.

Gilles Vassal, Villejuif, reported that the Cancer Drug Development Forum (CDDF), together with the Innovative Therapies for Children with Cancer Consortium (ITCC), the European Society for Paediatric Oncology (SIOPE) and the European Network for Cancer Research in Children and Adolescents (ENCCA), has created a unique Paediatric Oncology Platform, involving multiple stakeholders and the European Commission (EC), with an urgent remit to improve paediatric oncology drug development.

### Virtual tumour boards and transborder sarcoma therapy

Craig Gerrand, Newcastle upon Tyne, presented the UK ‘National Ewing’s Multidisciplinary Team’, which began as a pilot project in 2011 and serves as a forum to discuss the local treatment of patients with ES of bone. The aim was to improve the consistency of treatment decisions through discussion and peer review. In the 4 years since its establishment, over 295 patients have been discussed and the concept has been proven. More work is required to move beyond the pilot phase if the panel is to fulfil its potential, and support data collection and clinical trials in this and possibly other sarcoma types.

Stephanie Klco-Brosius, and Uta Dirksen Münster, presented the international interdisciplinary virtual EwS tumour board, which has been set up in Münster as an ENCCA project. Eight European and international countries discuss their patients in the virtual tumour board. A retrospective analysis was presented of the value of a reference tumour board, implicating major advantages for patients with primary disseminated disease.

Ruth Ladenstein, Vienna, reported on the European Expert Paediatric Oncology Reference Network for Diagnostics and Treatment (ExPO-r-Net) (http://www.expornet.eu). The main aim of ExPO-r-Net is to address and improve health inequalities for children with cancer in Europe by building a European Reference Network for Paediatric Oncology.

## Conclusion

Bone sarcomas are ultra orphan-diseases with three decades of stagnation in survival. To further improve outcomes, it is essential to promote international collaboration propelled by initiatives like the European Bone Sarcoma Network. Novel insights from basic and clinical research that may help to improve sarcoma therapy were discussed during the meeting and results were presented from investigations on tumour cell genomics, epigenetics, metabolism, altered signalling pathways, sarcoma immunology as well as on pharmacodynamics, pharmacogenomics, and the measurement of circulating tumour DNA. Moreover, experiences from recent sarcoma trials were reviewed and the viewpoints of patient and parent advocates, clinical researchers, statisticians and charity representatives were summarized in order to provide knowledge that can facilitate the design of future clinical sarcoma trials. The discussions generated many novel, exciting ideas, which now need to be taken forward to proposals for innovative trials, which are urgently needed. Such proposals for collaborative research and trials should be a major focus for the next European Bone Sarcoma Networking Meeting, scheduled to take place in London in 2017.

## Abbreviations

ABCB1: ATP-binding cassette transporter B1
ATM-CHEK1: aven-ATM serine/threonine kinase-checkpoint kinase 1
AXL: AXL receptor tyrosine kinase
BCOR: BCL6 co-repressor
BRCA1/2: breast cancer 1/2, early onset
BRD: bromodomain-containing proteins
CCNB3: cyclin B3
CDDF: cancer drug development forum
CDKN2A: cyclin-dependent kinase inhibitor 2A
CIC: capicua transcriptional repressor
CIK: cytokine-induced killer
COL2A1: collagen, type II, alpha 1
CRUK: cancer research UK
CS: chondrosarcoma
ctDNA: circulating tumour DNA
CTL: cytotoxic T-lymphocyte
CTU: clinical trials unit
DCC: DCC netrin-1 receptor
DUX4: double homeobox 4
EEA: European economic area
EEC: European Ewing consortium
EFACT: EWS-FLI sequence analysis from ctDNA
ELOVL5: ELOVL fatty acid elongase 5
ENCCA-WP7: European network for cancer research in children and adolescents-Work Package 7
EphA2: ephrin A receptor 2
ERCC1: excision repair cross-complementation group 1
ERG: v-Ets avian erythroblastosis virus E26 oncogene homolog
ES: Ewing sarcoma
EURAMOS: European and American osteosarcoma study group
EuroSARC: European clinical trials in rare sarcomas initiative
EWSR1: EWS RNA-binding protein 1
ExPO-r-Net: European expert paediatric oncology reference network for diagnostics and treatment
EXT1: exostosin glycosyltransferase 1
FEV: (FEV) ETS oncogene family
^18^F-FLT-PET/CT: 3′-deoxy-3′-^18^F-fluorothymidine positron emission tomograph/computed tomography
FLI1: Fli-1 proto-oncogene, FUS, FUS RNA binding protein
ETS: transcription factor
HDCHT: high-dose chemotherapy
HEY1: hes-related family BHLH transcription factor with YRPW motif 1
HGOS: high-grade osteosarcoma
HLA: human leukocyte antigen
IDH1: isocitrate dehydrogenase 1
IGF-1R: insulin growth factor 1 receptor
IMP: investigational medical product
IR: insulin receptor
ISG: Italian sarcoma group
ITCC: innovative therapies for children with cancer consortium
iTRAq: isobaric tags for relative and absolute quantification
LOH: loss of heterozygosity
MET: proto-oncogene, receptor tyrosine kinase
miRNA: micro RNA
MLS: myxoid/roundcell liposarcoma
mTOR: mammalian target of rapamycin
NAD: nicotinamide adenine dinucleotide
NAMPT: nicotinamide phosphoribosyltransferase
NCOA2: nuclear receptor coactivator 2
NFATc2: nuclear factor of activated T-cells, cytoplasmic, calcineurin-dependent 2
NGS: next-generation sequencing
NRAS: neuroblastoma RAS viral oncogene homolog
NTN1: netrin-1
PARP: poly ADP ribose polymerase
PATZ1: POZ (BTB) and AT hook containing zinc finger 1
PBSC: peripheral blood stem cells
PD-1: programmed cell-death 1
PD-L1: programmed cell-death ligand 1
PEA3: protein PEA3 (Ets variant 4)
PI3 K: phosphatidylinositol-4,5-bisphosphate 3-kinase
PMP22: peripheral myelin protein 22
PROVABES: prospective validation of biomarkers in Ewing sarcoma network
RANK: receptor activator of nuclear factor-kappa B
RB: retinoblastoma
RNA-Seq: RNA sequencing
RON: recepteur d’origine nantais (macrophage stimulating 1 receptor)
RTK: receptor tyrosine kinase
SIOPE: European Society for Paediatric Oncology
siRNA: small interfering RNA
SARC: Sarcoma Research Through Collaboration
SNP: single-nucleotid polymorphism
SPAEN: Sarcoma patients EuroNet
SSG: Scandinavian sarcoma group
TP53: tumour protein 53
TRAP: tartrate-resistent acid phosphatise
UNC5H: Unc-5 homolog C
VEGFA: vascular endothelial growth factor A

